# Measures to Promote Rural Healthcare Tourism with a Scientific Evidence-Based Approach

**DOI:** 10.3390/ijerph17093266

**Published:** 2020-05-07

**Authors:** Dawou Joung, Bohwi Lee, Jeongdo Lee, Changjun Lee, Seungmo Koo, Changwon Park, Sebin Kim, Takahide Kagawa, Bum-Jin Park

**Affiliations:** 1 Department of Environment and Forest Resources, Chungnam National University, 99 Daehak-ro, Yuseong-gu, Daejeon 34134, Korea; dawo.jeong@gmail.com (D.J.); bohwi00@gmail.com (B.L.); volom19@gmail.com (J.L.); lcj3529@naver.com (C.L.); sbkim@cnu.ac.kr (S.K.); 2 Department of Agricultural Economics, Chungnam National University, 99 Daehak-ro, Yuseong-gu, Daejeon 34134, Korea; koosm@cnu.ac.kr; 3 Gyeonggi Regional Headquarter, Korea Rural Community Corporation, 347 Jangan-ro, Jangan-gu, Suwon-si, Gyeonggi-do 16346, Korea; pcwhome@ekr.or.kr; 4 Forestry and Forest Products Research Insitute, 1 Matsunosato, Tsukuba, Ibaraki 305-8687, Japan; kagawa@ffpri.affrc.go.jp

**Keywords:** healthcare tourism, forest bathing, rural and mountain economy, physical activity, walking, heart rate variability, salivary cortisol, POMS, STAI

## Abstract

The present study aimed to evaluate the effects of physical activities on human health in forests in countryside and rural areas. The test experiment was conducted in a countryside forest, whereas the controlled experiment was conducted in an urban area where the study participants resided. A total of 22 participants (aged 20.9 ± 1.3 years) were evaluated in this study. Heart rate variability and salivary cortisol level were used as indices of physiological conditions, and semantic differential method, profile of mood states (POMS), and state-trait anxiety inventory (STAI) were used to evaluate the participants’ emotional states. The participants were asked to walk around forest and urban areas for 15 min. The results were as follows. As compared to the urban area, in the forest area, (1) the power of the high-frequency (HF) component of the heart rate variability (HRV) was significantly higher; (2) low-frequency (LF)/(LF + HF) was significantly lower; (3) salivary cortisol level was significantly lower; (4) the participants felt more comfortable, natural, relaxed, and less anxious and showed higher levels of positive emotions and lower levels of negative emotions. Consequently, walking in the forest area induces relaxing short-term physiological and psychological effects on young people living in urban areas.

## 1. Introduction

In the last several decades, due to compressive industrialization and modernization after the 1960s, Korea has achieved a rapid economic growth [1]. However, after its economies increased, agricultural and mountain villages have undergone demographic changes, such as aging and over-depopulation [2]. According to the 2015 Agriculture, Forestry & Fishery Census Report released by Statistics Korea, between 2000 and 2015, the proportion of the farming population aged over 65 years increased from 14.7% to 38.4% [3]. This is approximately three times higher than the proportion of the total Korean elderly population [3]. If these demographic trends continue in agricultural and mountain villages, the villages will fail to perform their roles as local communities and will eventually become extinct. This would also lead to the loss of capability to exploit the ecosystem services offered by rural and mountainous areas, such as food growth and recreation.

To solve this demographic problem, until the 1990s, the Korean government pursued policies for the development of agricultural and mountain villages, focusing on regional infrastructure maintenance (community facilities and housing) [4]. Since the 1990s, as stagnant farm incomes accelerated the income gap among farming communities, the Korean government paid extra attention to increasing non-agricultural incomes [5].

Previous studies [6,7,8] have reported that, although primary industries, such as forestry and agriculture are declining, tourism becomes a tool to help creating local jobs and raising the level of economic welfare. Based on these studies, the Korean government has used more regional resources and pursued policies targeting urban–rural coordination and regional synergies, encouraging community participation, and focusing on rural, green, and eco-tourism [9]. As part of national projects, Korean Forest Service has designated some countryside forests, approved by an assessment procedure, as “Healing forest” [10]. Such “healing forest” generally provides visitors with rural tourism in Korea and is also representative of the forest-to-healthcare business. Activities in healing forests reportedly reduce healthcare expenditure by 70,000 Korean won (62 dollars) per person and generate approximately 1.4 trillion Korea won (126 million dollars) in economic value, including approximately 20,000 additional job opportunities [11].

Recently, interest in ecology and health has continued to grow worldwide, and a number of studies have reported that natural and green spaces promote modern human health [12,13,14,15,16,17,18,19,20,21,22,23,24]. According to these studies [12,13,14], direct contact with nature, such as viewing natural landscapes or walking in the forest, increases parasympathetic nervous activity and suppresses sympathetic nervous activity. Previous studies [15,16] also reported a decrease in cerebral blood flow and oxyhemoglobin concentration in prefrontal cortex activity. It was reported that green environment reduces the levels of salivary cortisol, a stress hormone [12,15,17]. Short stays in the forest also strengthen natural killer cell activity and improve immune system, and this effect can last for approximately 30 days [18,19,20,21]. In addition, negative emotions such as anxiety, depression, and tension are reduced, while positive emotions increase, and psychological relaxation effects are enhanced [14,16,22,23,24]. These relaxing effects of forest are produced by gaining information about the physical environment, such as air temperature, humidity, illuminance, sounds, etc., as well as about chemical environments such as phytoncides of forest through our five senses [25,26]. The phytoncides are volatile organic compounds derived from trees [25]. Alpha-pinene and d-limonene of monoterpenes are known as the common components of phytoncides [26,27] and were reported to have psychological and physiological relaxing effects [28,29]. Meanwhile, the phytoncides are known to be highly emitted from coniferous trees, such as *Chamaecyparis obtusa*, *Cryptomeria japonica*, and *Pinus koraiensis*. Among these, *Chamaecyparis obtusa* was reported to emit a relatively larger total amount of phytoncides as compared to other species [30]. In addition, volatile essential oils from *Chamaecyparis obtusa* were reported to provide physiological relaxing effects [31], immunity improvement [32], and antibacterial and antifungal effects [33]. Based on such scientific evidence, South Korea is trying to revitalize the rural tourism aimed at health care through the forest resources, which account for 63% of the nation’s total land [34].

Rural tourism is a multi-faceted activity occurring in the countryside and it includes walking, climbing, adventure, and hunting [35]. Walking is the most common form of physical activity [36]. Walking requires no special equipment or clothing, is very simple, and can be carried out at any place [37]. It is also associated with a lower risk of injury involved in other physical activities [38]. It is known to have beneficial effects on risk factors associated with cardiovascular diseases, including blood pressure, obesity, cholesterol, and diabetes [39]. These facts mean that walking is suitable as an activity of rural health tourism.

In order to promote the rural health tourism, it is necessary to clarify the health promotion effects of rural visits. Therefore, the present study aimed to evaluate the effects of walking in forests in rural areas on human health. The hypotheses tested in the present study are as follows:

1. Short-term walking in the forest in rural areas will increase parasympathetic nervous activity.
2. Short-term walking in the forest in rural areas will decrease sympathetic nervous activity.
3. Short-term walking in the forest in rural areas will reduce salivary cortisol levels.
4. Short-term walking in the forest in rural areas will reduce negative emotions (i.e., anxiety, depression, anger, fatigue, and confusion).
5. Short-term walking in the forest in rural areas will make one feel more comfortable, natural, and relaxed.

## 2. Materials and Methods

### 2.1. Participants

Study participants were college students in their 20s who were urban residents and who volunteered to participate in the study. They were recruited through advertisements posted on campus bulletin board, social network service, and website. Exclusion criteria included smoking or taking any medications. The experiment was performed among a total of 24 healthy college students (15 males and 9 females, aged 20.8 ± 1.3 years). All participants were fully informed about the experimental procedures and methods prior to participating in the experiment. The research was conducted according to the guidelines of the Declaration of Helsinki, and all experimental procedures were reviewed and approved by the Research and Bioethics Committee of Chungnam National University.

### 2.2. Study Area

This study was conducted in the forest area of Chukreong Mountain and the Daejeon metropolitan city as an urban area.

**Figure 1:**
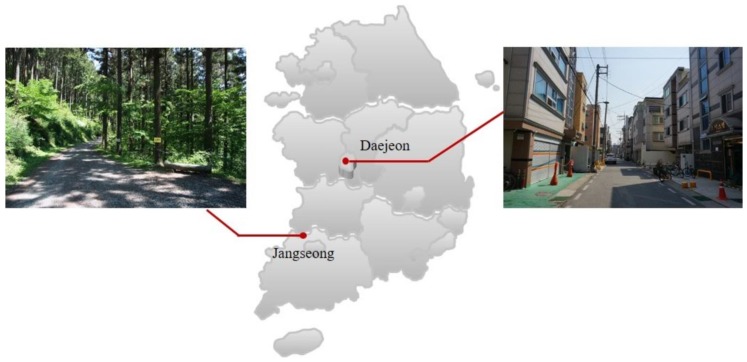
Location of the study areas and the scenery at the two experiment sites.

The Chukreong Mountain is located in Seosam-myeon, Jangseong-gun, Jeollanam-do, Republic of Korea (Figure 1). The Chukreong Mountain (1148 ha) is set in the largest warm-temperate forest in Korea and is full of evergreen trees, such as *Chamaecyparis obtusa* (Siebold & Zucc.) Endl. and *Cryptomeria japonica*. This mountain comprises six walking trails (of 10.8 km in length in total) and rural tourism villages, which offer overnight accommodation and recreational activities. The Korean government designated some forests as healing forests, with themes of well-being, wellness, and relaxation, under the motto of “Welfare-to-Forest”.

Some parts of Chukreong Mountain are designated as a healing forest and “national healing forest of Jangseong” and various health-enhancing activities using the fragrant smell of the forest and landscapes are available for visitors.

The control site was an urban area in Gung-dong, Yuseong-gu, Daejeon metropolitan city, Republic of Korea (Figure 1). We selected urban areas where the participants actually reside to reflect the daily lives of urban people.

The forest walking course was selected in the forest zone of *Chamaecyparis obtusa*. The urban walking course was selected in the residential areas among houses and stores with few trees. All the selected walking courses were almost flat.

### 2.3. Physiological Markers

#### 2.3.1. Heart Rate Variability (HRV)

HRV is a reliable measure of autonomous nervous system functioning using R–R intervals in heart rate (thereafter referred to as RRI) [40,41]. In this study, RRI was measured using a portable electrocardiogram (Activtracer AC-301A, GMS, Japan). The frequency analysis of RRI was performed using the maximum entropy method (Memcalc/win; GMS, Japan). The low-frequency (LF) range was set from 0.04 to 0.15 Hz, whereas the high-frequency (HF) range was set from 0.15 to 0.4 Hz [40].

The HF component was used as an index of parasympathetic nervous system activity, which is typically more active during relaxation. Higher HF values indicate a greater parasympathetic nervous system activity. The LF/(LF + HF) value was used as an index of sympathetic nervous system activity, which is typically enhanced in hypertension [40,42]. LF/(LF + HF) means normalized LF band, the normalized measure was a computed index that was not directly estimated from the raw RRI data itself, but was computed as a second step after the initial statistical estimation of the power in the LF and HF components of the HRV spectrum [43]. Lower LF/(LF + HF) values indicate a decrease in sympathetic nervous system activity.

#### 2.3.2. Salivary Cortisol Concentration

Cortisol, a typical stress hormone, has been widely used as an index of stress levels [44], and its levels can be measured in blood or saliva. The collection of saliva has the advantage of being noninvasive, making sampling easy and stress-free, and it can be performed at any time and at any place. In the present study, cortisol measurements were performed using saliva samples. Cortisol has its distinct circadian rhythm, which is a 24 h cycle [45]. Therefore, saliva samples were collected from the participants in the forest and urban areas at the same time point in this study. The saliva samples were collected using the Salivette^®^ (Sarstedt, Germany) system. The Salivette^®^ kit comprises a centrifuge vessel, a suspended insert, and a swab stopper. The swab from the kit was placed in each participant’s mouth, and approximately 2 mL of saliva were collected over 2 min. The kit was then frozen and tested for the quantitative measurement by Samkwangmedical laboratories, Republic of Korea, using enzyme immunoassay (EIA).

### 2.4. Questionnaires

After completing the walks, the participants completed three types of psychological questionnaires that asked them about their emotions, mood, and anxiety.

#### 2.4.1. Semantic Differential (SD) Method

The SD method [46] is widely used to evaluate scenery, a factor that is difficult to quantify owing to subjective differences. The study evaluated feelings on a 13-point scale using adjectives in Korean, such as “comfortable–uncomfortable”, “natural–artificial”, and “soothed–stimulating”. The Cronbach’s alpha in this study was 0.926.

#### 2.4.2. Profile of Mood State

The profile of mood state (POMS) was used to evaluate psychological reactions. This study used a shortened version of POMS, which is a method to evaluate respondents’ emotions, such as “tension and anxiety”, “depression”, “anger and hostility”, “vigor”, “fatigue”, “confusion”, and “total mood disturbance (TMD)” using 30 questions [47,48]. TMD scores were calculated to denote an overall assessment of emotional state. We used the Korean version of POMS [49]. The Cronbach’s alpha in the present study was 0.918.

#### 2.4.3. State-Trait Anxiety Inventory (STAI-X)

STAI-X [50] is a self-assessment questionnaire that measures personal subjective feelings, such as tension, apprehension, worry, and nervousness. The questionnaire comprises 20 items enquiring to what extent respondents are currently experiencing the symptoms or signs: “not at all”, “somewhat”, “moderately”, or “very much”. These items are rated on a 4-level scale, with a possible score ranging from 20 to 80. Higher scores indicate more anxiety. We used the Korean version of STAI-X [51]. The Cronbach’s alpha in this study was 0.919. Meanwhile, despite the existence of “anxiety” as a sub scale of the POMS, in order to increase the reliability of the study results, STAI was used.

### 2.5. Procedure

Field experiments were conducted in both forest and urban areas. The experiments were designed using the single-group crossover design [52]. The physiological and psychological relaxing effects of forests were determined following previous studies [12,13,14,15,16,17,18,19,20,21,22,23,24]. To avoid any influence of the relaxing effect of forests on the outcome of the controlled experiments, all participants were asked to walk around urban areas before forest areas. The participants visited an urban area in Yuseong-gu, Daejeon as the controlled experiment, on 2 May 2014. They also visited “Jangseong healing forest” located at Jangseong-gun, Jeollanam-do, on 6 May 2014.

**Figure 2:**
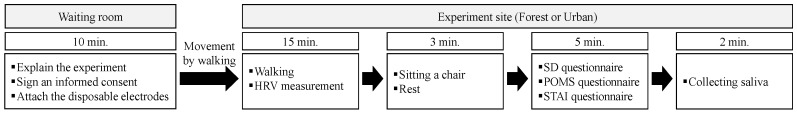
Experimental design.

Each experiment was conducted during a one-day field trip in sunny weather. All procedures of the experiment were conducted identically. The procedures are described in Figure 2.

First, a waiting room was set up for the participants near the starting point of each walking course. In this room, all participants were informed about the experiment while they were waiting. Electrodes were then attached to their skin in this room. All participants walked for 15 min along each course at the same speed. The participants were asked to walk slowly, viewing the landscape of the given area. To obtain electrocardiogram (ECG) data, three disposable electrodes were attached to their chests before walking. ECG data were sequentially recorded by a portable electrocardiogram (Activtracer AC-301A, GMS, Japan) while the participants were walking each course. After completing the walk, all participants then rested for approximately 3 min by sitting on chairs and filled in the three questionnaires (SD method, POMS, and STAI).

Salivary cortisol levels were measured at the end of the experiment to avoid the influence caused by any form of physical activity. To measure the cortisol levels in saliva, absorbent cotton for Salivette^®^ (Sarstedt, Germany) was placed in the participants’ mouths, and saliva samples were collected for 2 min.

### 2.6. Data Analysis

The data were analyzed for a total of 22 participants; 2 participants were excluded from the analysis (one consumed alcohol during the experiment, and the other was a dropout). All statistical analyses were performed using Statistical Package for Social Sciences software version 21 (IBM Corp., Armonk, NY, USA). A paired *t*-test with Holm’s correction was used to compare the differences in physiological responses for 1 min during walking in the forest and urban areas. Wilcoxon signed-rank test was used to analyze the differences in psychological responses between forest and urban areas. A one-sided test was used in this study. The significance level was set at *p* < 0.05.

## 3. Results

### 3.1. Physiological Markers

**Figure 3:**
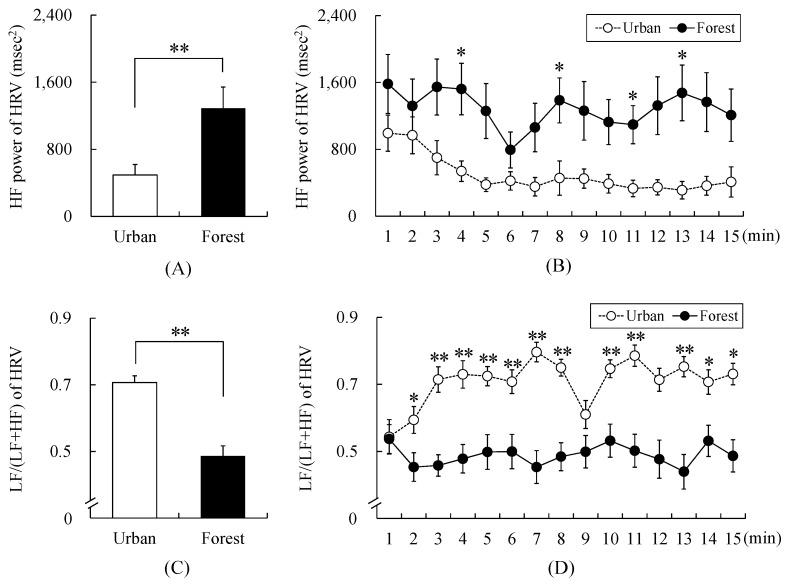
Change in heart rate variability (HRV) while walking in the forest and urban areas. (**A**) Change in high-frequency (HF) power while walking in the forest and urban areas, (**B**) Temporal changes in HF power while walking in the forest and urban areas, (**C**) Change in the low-frequency (LF)/(LF + HF) power while walking in the forest and urban areas, (**D**) Temporal changes in the LF/(LF + HF) value while walking in the forest and urban areas. Data are presented as mean ± standard error. Significant differences verified by paired *t*-test with Holm’s correction was used; * *p* < 0.05 and ** *p* < 0.01.

There was a significant difference in the change in HRV and cortisol levels between walking through forest and urban areas. The HF values, an index of parasympathetic nervous system activity, were 1290.71 ± 249.90 and 493.58 ± 124.46 ms^2^ in the forest and urban areas, respectively. The HF values of the forest area were by 161.50% higher than that of the urban area (*p* < 0.01; Figure 3A). After an analysis was performed at 1-min interval, the HF components were higher in the forest area than in the urban area. Specifically, it was significantly higher at four different time points (4, 8, 11, and 13 min) (*p* < 0.05; Figure 3B).

The values of LF/(LF + HF) of HRV, which is an index of sympathetic nervous system activity, were 0.49 ± 0.03 and 0.71 ± 0.02 in the forest and urban areas, respectively. The LF/(LF + HF) value of the forest area was 30.99% lower than that of the urban area (*p* < 0.01; Figure 3C). An analysis was performed at 1-min interval, and the results showed that the LF/(LF + HF) value was lower in the forest area than in the urban area, such that it was significantly lower at 2–8- and 10–15-min intervals (*p* < 0.05; Figure 3D).

**Figure 4:**
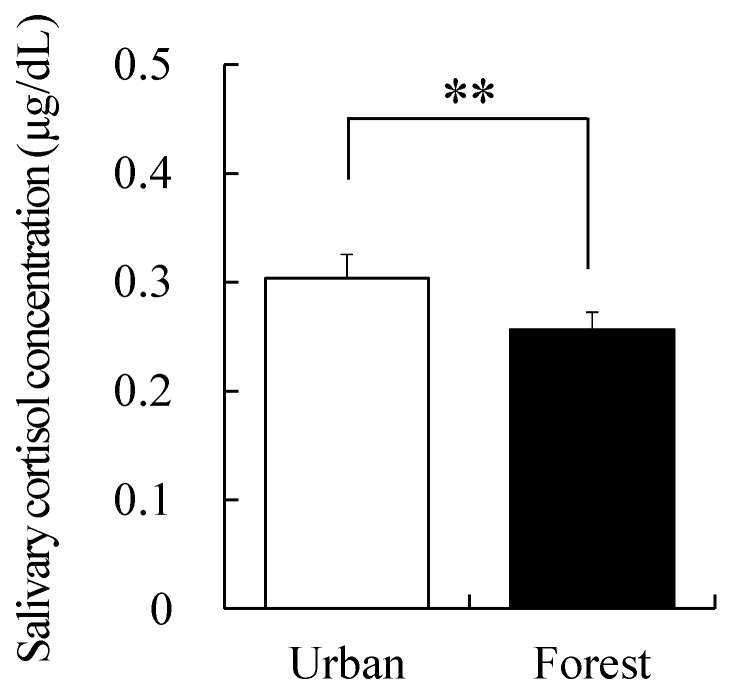
Comparison of the salivary cortisol levels after walking in the forest and urban areas. Data are presented as mean ± standard error. Significant differences verified by paired *t*-test were used; ** *p* < 0.01.

A significant difference was also observed in the cortisol levels in saliva, which is an index of stress. Cortisol levels were 0.26 ± 0.0 µg/dL in the forest area, whereas it was 0.30 ± 0.0 µg/dL in the urban area, indicating that cortisol levels were by 13.33% lower in the forest area than in the urban area (*p* < 0.01; Figure 4).

### 3.2. Questionnaires

**Figure 5:**
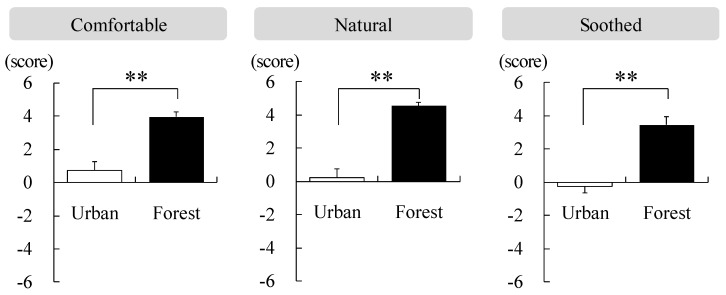
Comparison of the feelings (comfortable, natural, and soothed) while walking in the forest and urban areas. Data are presented as mean ± standard error. Significant differences verified by Wilcoxon signed-rank test was used; ** *p* < 0.01.

Using the SD method, an investigation was performed to measure the participants’ feelings of comfort, nature, and relaxation in the forest and urban areas. With regard to comfort, the method revealed 3.95 ± 0.28 points in the forest area and 0.73 ± 0.52 points in the urban area. Regarding the feeling of nature, the method revealed 4.55 ± 0.22 points in the forest and 0.23 ± 0.54 points in the urban area. Regarding the feeling of relaxation, it was 3.45 ± 0.39 points in the forest and −0.27 ± 0.50 points in the urban area. The participants felt more comfortable, natural, and soothed in the forest area than they felt in the urban area (*p* < 0.01; Figure 5).

**Figure 6:**
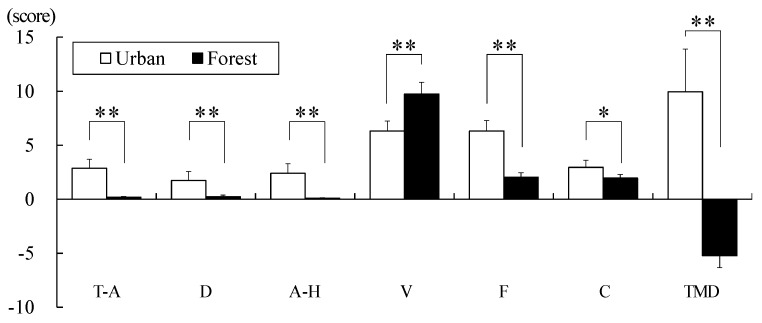
Comparison of the profile of mood states (POMS) while walking in the forest and urban areas. T–A: tension-anxiety; D: depression-dejection; A–H: anger-hostility; V: vigor; F: fatigue; C: confusion; TMD: total mood disturbance. Data are presented as mean ± standard error. Significant differences verified by Wilcoxon signed-rank test was used; * *p* < 0.05 and ** *p* < 0.01.

The participants’ mood state in the forest and urban areas was investigated using POMS. The results showed that the participants’ negative mood states, such as tension, depression, anger, fatigue, and confusion, were significantly lower in the forest area than in the urban area. In comparison, the positive mood states, such as vigor, were significantly higher in the forest area than in the urban area. The TMD score was −5.23 ± 1.09 points in the forest area, whereas it was 9.95 ± 3.95 points in the urban area. The TMD scores were significantly lower in the forest area than in the urban area (*p* < 0.01, Figure 6), indicating that the participants had more negative mood states in the urban area than in the forest area.

**Figure 7:**
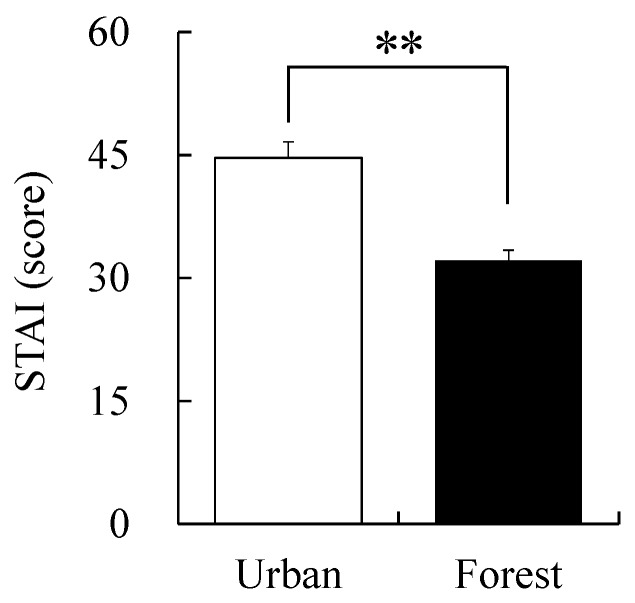
Comparison of the state-trait anxiety inventory (STAI) while walking in the forest and urban areas. Data are presented as mean ± standard error. Significant differences verified by Wilcoxon signed-rank test was used; ** *p* < 0.01.

STAI revealed that the state of anxiety was 32.14 ± 1.26 points in the forest area, whereas it was 44.64 ± 1.97 points in the urban area. The anxiety that the participants experienced in the forest area was 28.00% lower than that in the urban area (*p* < 0.01, Figure 7).

## 4. Discussion

The study aimed to evaluate the short-term effects of walking in forests in rural areas on young people living in urban areas and to lay the groundwork for rural tourism with scientific evidence-based healthcare approach in mountain villages.

Compared with walking in an urban area, walking in a forest area significantly increased the HF component, which is an index of parasympathetic nervous activity, and significantly decreased the LF/(LF + HF) value of HRV, which is an index of sympathetic nervous activity. These results are consistent with the previous findings that walking in the forest provides relaxation to the autonomic nervous system [12,13,14]. The levels of cortisol, a stress hormone, were significantly lower in the forest area in rural area than in the urban area. This result supports previous research that demonstrated that physical activity in forests reduces stress hormones [12,15,17].

The results of psychological evaluations showed that forest environment can promote psychological relaxing effects. Walking in the forest in a rural area enhanced the participants’ feelings of comfort, naturalness, and feeling soothed, as well as reduced their negative emotions, such as tension–anxiety, depression, anger–hostility, and confusion. These results are consistent with those reported in previous studies [14,16,22,23,24].

The findings of the present study strongly support the biophilia hypothesis [53], psycho-evolutionary theory [54], and attention restoration theory [55], according to which nature has a positive impact on human health.

According to the attention restoration theory (ART), a period of prolonged mental effort leads to directed attention fatigue [55,56]. It can be recovered through environments that do not require directed attention [57]. The restorative environment proposed in ART refers to an environment that satisfies four factors: being away, fascination, extent, and compatibility [55,56,58]; according to Kaplan [56], the natural environment satisfies these factors. A number of previous studies [12,13,14,15,16,17,18,19,20,21,22,23,24,59,60] based on the ART proved that nature is suitable for a recovery environment. The results of this study scientifically revealed that the forests in rural areas, which are part of nature, can be effectively used as a recovery environment for people living in urban areas.

Previous research suggested that quality is an important criterion of a green space [61]. The quality of green areas can be defined in terms of biodiversity [62]. Green areas on the outskirts of cities and high biodiversity green areas are more likely to exert restorative effects on their visitors [62]. Urban forests, and sometimes even more forests on the outskirts of urban areas, could even worsen the air quality due to high concentration levels of nitrogen oxides, leading to the formation of secondary organic aerosols and ozone [63,64]. Nevertheless, urban areas still have more serious air pollution than rural areas [65], and people living in urban areas experience a higher exposure to air pollution than those living in rural areas [66]. Meanwhile, forests eliminate the substantial amounts of air pollution and can produce substantial health benefits [67]; overall, pollution removal is substantially higher in rural areas than in urban areas [68]. These results imply that rural areas with relatively high species diversity and low air pollution levels may be more conducive to improving health.

The integration of rural tourism with healthcare activities can solve the problem of modern people who suffer from continuous stress, thereby improving their physical, mental, and social health, and can help in overcoming the constraints of mountain villages. Promoting the health-enhancing effects of rural healthcare tourism is the key to help people understand rural healthcare tourism better and encourage their participation. The results of the present study provide scientific evidence in support of this conclusion.

The present study has several limitations. First, we could not check the respiration rate of the participants. Respiration rate can affect measurements of HRV from the frequency domain [69]. Therefore, when using HRV as an indicator, there is a need to consider the respiration rate. Second, all participants were aged in their 20 s, so our results demonstrate the potential value of forests as health care providers to younger generations living in urban areas. However, to be able to generalize the results of the study, there is a need for further research including individuals from various age groups. Third, it cannot be excluded that phytoncides had affected the participants’ relaxation effects. Therefore, follow-up studies need to investigate the concentration or emission patterns of phytoncides, and to study whether it affects the relief effect of short-term walking in forest areas. Meanwhile, the concentration of phytoncides shows extremely high levels of variability in time and space, along with important regularity [70]. Therefore, further research would need to map and predict the concentration of effective phytoncides. Doing so would allow planning a stay in the forest and optimizing the benefits, as well as would be an additional attracting service to the visitors.

The results of this study clearly revealed the relaxing effects of a short-term walk in the forest in a rural area. However, we do not know how long the effects would last. Therefore, follow-up studies that would estimate the duration of the effects are needed. For the mutual prosperity of mountain villages and urban areas, long-term projects need to be conducted to establish a well-organized database of scientific evidence based on forest resources.

## 5. Conclusions

The results of this study indicate that, as compared to walking in an urban area, walking in a forest area increases parasympathetic nervous activity, decreases sympathetic nervous activity, and reduces cortisol levels in saliva. Moreover, walking in a forest area was found to make the study participants feel more comfortable, natural, relaxed, and vigorous, as well as to experience less anxious and negative emotions, as compared to walking in an urban area. In conclusion, walking in a forest area induces short-term relaxing physiological and psychological effects on young people living in urban settlements.
